# Effect of Cold Storage and Reheating of Parboiled Rice on Postprandial Glycaemic Response, Satiety, Palatability and Chewed Particle Size Distribution

**DOI:** 10.3390/nu9050475

**Published:** 2017-05-10

**Authors:** Louise Weiwei Lu, Bernard Venn, Jun Lu, John Monro, Elaine Rush

**Affiliations:** 1 School of Sport and Recreation, Faculty of Health and Environmental Sciences, Auckland University of Technology, Auckland 1010, New Zealand; elaine.rush@aut.ac.nz; 2 Human Nutrition Unit (HNU), School of Biological Sciences, University of Auckland, Auckland 1010, New Zealand; 3 Department of Human Nutrition, University of Otago, Dunedin 9016, New Zealand; Bernard.venn@otago.ac.nz; 4 School of Science, and School of Interprofessional Health Studies, Faculty of Health and Environmental Sciences, Auckland University of Technology, Auckland 1010, New Zealand; jun.lu@aut.ac.nz; 5 The New Zealand Institute for Plant & Food Research, Palmerston North 4474, New Zealand; john.monro@plantandfood.co.nz

**Keywords:** parboiled rice, medium-grain white rice, cold-stored, reheating, blood glucose concentration chewing time, satiety, palatability

## Abstract

**Background:** Globally, hot cooked refined rice is consumed in large quantities and is a major contributor to dietary glycaemic load. This study aimed to compare the glycaemic potency of hot- and cold-stored parboiled rice to widely available medium-grain white rice. **Method**: Twenty-eight healthy volunteers participated in a three-treatment experiment where postprandial blood glucose was measured over 120 min after consumption of 140 g of rice. The three rice samples were freshly cooked medium-grain white rice, freshly cooked parboiled rice, and parboiled rice stored overnight at 4 °C. All rice was served warm at 65 °C. Chewing time was recorded. **Results**: incremental area under the curve (iAUC) of the control rice, freshly cooked medium-grain white rice, was the highest: 1.7-fold higher (1.2, 2.6) than reheated parboiled rice (*p* < 0.001) and 1.5-fold higher (1.0, 2.2) than freshly cooked parboiled rice (*p* = 0.001). No significant difference in postprandial glycaemic response was observed between freshly cooked and reheated parboiled rice samples (*p* = 0.445). Chewing time for 10 g cold-stored parboiled rice was 6 s (25%) longer and was considered more palatable, visually appealing and better tasting than freshly cooked medium-grain (all *p* < 0.05). **Conclusions**: For regular consumers of rice, reheating cooked rice after cold storage would lower the dietary glycaemic load and, in the long term, may reduce the risk for type 2 and gestational diabetes. More trials are needed to identify the significance.

## 1. Introduction

Globally, the consumption of rice, which currently provides the majority of daily energy intake and carbohydrate for at least half of the world’s population [1], has been increasing. Since the Joint FAO/WHO Expert Consultation on Carbohydrates in Human Nutrition in April 1997 [2], there has been increased understanding of the diverse physiological roles that carbohydrates have on the rate and extent of digestion in the gut and the relationship between dietary carbohydrates and various non-communicable diseases, including hyperglycaemia, insulin resistance, obesity, metabolic syndrome and type 2 diabetes [3].

The metabolic quality of carbohydrate sources such as rice may be directly determined by the effect on biomarkers of carbohydrate metabolism, such as glycaemic response (i.e., postprandial blood glucose concentration). The key determinants of the postprandial blood glucose response are the amount, rate and extent of carbohydrate digestion [4], insulin secretory response [5] and gastric emptying [6,7]. The extent of digestion, and thus the glycaemic response, are determined by the particle size of the food, the cooking method, the size of the mouthful, the extent of chewing and digestion that takes place in the mouth, and the physical and chemical properties of the starch [8,9].

One measure of a healthier cooked rice meal would be reduction of the glycaemic load (GL, i.e., slower and lower sustained release of glucose) by providing a lower proportion of rapidly digestible starch (RDS, starch that can be digested within 20 min following ingestion). This means higher proportions of slowly digestible starch (SDS, starch that can be digested between 20 and 180 min following ingestion) and resistant starch (RS, starch that can resist digestion for up to 180 min following ingestion).

Cooling cooked rice to low temperatures by refrigeration transforms gelatinised rice starch from an amorphous state to a more ordered state (i.e., crystalline state) that persists on reheating. This crystallised starch form can resist enzymatic degradation in the small intestine for up to three hours [10] and spontaneously lower the concentration of digestible starch in cooked rice [11]. Previous in vitro digestion studies have also shown that freshly cooked warm rice was digested more rapidly than cold-stored and reheated rice and minced-reheated parboiled rice was more resistant to digestion [12]. Three previous studies, by Wolever [13], Larsen [14], and Chitra [15], have investigated the commercially obtained parboiled rice products (processed by modernised parboiling process) and found lower glycaemic response in the freshly prepared parboiled rice samples compared with non-parboiled, but satiety was not measured in these studies. Furthermore, the glycaemic response of cooked parboiled rice after the cold storage and reheating process has not been investigated.

Rice grains are consumed as whole, therefore, the particle size reduction solely depends on mastication habit (i.e., chewing time and style). Mastication habits vary widely among individuals but show relatively consistent patterns within an individual when a single food has been tested [16]. Previous in vitro experiments [12] and an in vivo study [16] reported that particle size decrease exposes more surface area to digestive enzymes, resulting in an increase in the rate of digestion. The effect of habitual mastication and the degree of particle size reduction on glucose responses of a single food has been investigated in previous studies [16,17,18]. The degree of particle size reduction and its impact on glucose response among rice varieties have not been investigated previously.

It was hypothesised that cooking followed by cold storage at 4 °C for 24 h and subsequent reheating to 65 °C for 15 min, in accordance with safe practice [19], would result in parboiled rice inducing a significantly lower glycaemic response than both freshly prepared medium-grain white rice and parboiled rice. A secondary hypothesis was that the cold storage and reheating process would significantly increase the chewing time, reduce the chewed particle size, and increase the glycaemic response. Moreover, consumption of reheated parboiled rice would enhance satiety and reduce palatability.

## 2. Materials and Methods 

### 2.1. Study Design

Twenty-eight healthy subjects participated in a cross-over study randomised for order of the three treatments on three different days. The three treatments were freshly cooked medium-grain white rice (Control, Oryza sativa japonica, Australia imported raw medium-grain white rice (SunRice^®^, Leeton, Australia)), freshly cooked parboiled rice (Test Rice 1, Oryza sativa indica, produced and imported from Thailand (RealRice^®^, Thailand imported)), and reheated parboiled rice (Test Rice 2, Oryza sativa indica, produced and imported from Thailand (RealRice^®^, Thailand imported)). The primary outcome was postprandial blood glucose concentration trajectory. Secondary outcomes were chewing time, chewed particle size distributions, satiety and palatability responses. The design of the primary experiment was based on glycaemic index (GI) methodology but used a one-cup serving of rice [20] to reflect the quantity of rice usually eaten rather than a standard dose of carbohydrate.

The required sample size was estimated based on the review by Venn and Green [21] and the rice GI data from the Sydney University GI database. To detect a 20% reduction in postprandial blood glucose incremental area under the curve (iAUC) among differences between rice samples, and setting α-error to 0.05 and β-error to 0.90, it was estimated that 25 participants were required if each rice sample was tested once. Similarly, based on several previous satiety investigations using visual analogue scales (VAS) [22] it was determined that to achieve a practically significant difference in satiety of 20% with 80% power would require a sample size of 13 participants.

### 2.2. Participants

Adult participants were recruited from the Dunedin, New Zealand, as a convenient sample. Volunteers were recruited using advertisements, flyers, information sheets, notices and Internet postings and assessed for eligibility using an online survey (Survey Monkey, Palo Alto, CA, USA). Inclusion criteria included age between 18 and 45 years living in Dunedin. Exclusion criteria included self-reported smoker, pregnancy, being diagnosed with long-term illness (i.e., metabolic disorder, cancer, cardiovascular diseases), currently on long-term medication, and having abnormal fasting blood glucose (fasting finger prick blood glucose over 6.0 mmol/L [23]). Eligible persons were screened for body mass index (BMI): heights (m) and weights (kg) were measured using Segmental Body Composition Analyser (BC-418, Tokyo, Japan). Volunteers with BMI over 25.0 kg/m^2^ were categorised as overweight or obese, and between 18.0 and 25.0 kg/m^2^ as normal. To control for body mass index (BMI) effects on glycaemic responses and to broaden generalisability, around half of the participants were screened for normal weight and half for being overweight or obese (WHO criteria). All volunteers were screened for fasting finger prick glucose (using a HemoCue reader, HemoCue^®^ Hb 201 System, Ängelholm, Sweden). Thirty participants were eligible and recruited. Two participants later withdrew due to other commitments.

This study was conducted according to the guidelines of the human ethics committees of the University of Otago and the Auckland University of Technology (AUT). Both university ethics committees approved all procedures involving human participants. Ethical approval for the study was obtained from the University of Otago Ethics Committee (12/333) and the AUT Ethics Committee (EA13/05).

### 2.3. Rice Treatments

Three 1 kg bags of medium-grain white and two 2 kg bags of parboiled long-grain rice were purchased from New Zealand Dunedin supermarkets. Both rice products were from the same batch to avoid inter-batch variation. The rice was cooked following the instructions provided by the rice manufacturers to best reproduce the habitual cooking procedure. The rice-to-water ratio was 3:4.5 for medium-grain white rice using standard measuring cups (Farberware^®^ Classic Measuring Cup, 1 cup = 200 g rice or 250 mL of tap water) and 3:7 for parboiled rice. Rice samples were cooked to full gelatinisation in an automatic rice cooker (Tefal^®^ R07, Rumilly, France) at room temperature. The freshly cooked medium-grain rice (Control) and freshly cooked parboiled rice (Test Rice 1) were checked for temperature (around 65 °C) and then served to participants in preheated bowls within 10 mins following cooking.

For cold-stored and reheated parboiled rice (Test Rice 2), the rice sample was firstly cooked to full gelatinisation as for Test Rice 1. Approximately 140 g of freshly cooked parboiled long-grain white rice was weighed using electronic scales (Sartorius^®^, CP4202S, Goettingen, Germany) and placed into a shallow bowl (4 cm deep, pre-cooled to 4 °C in the refrigerator) to ensure instant cooling. Plastic food wrap was wrapped around the bowl to prevent moisture loss. The sealed rice bowls were placed in the refrigerator for rapid cooling to 4 °C and for 24-h storage. The temperature of the cooled rice was checked three times: at three hours, six hours and twenty four hours after refrigeration, using a clean wiped thermometer. Before consumption, the bowls were removed from the refrigerator and reheated in a convection microwave oven (Sharp^®^, R99, Osaka, Japan) at 1000 W power until the temperature after stirring reached 65 °C.

### 2.4. Study Visits

Participants were randomly allocated a rice sample of either Control Rice, Test Rice 1, or Test Rice 2 in a repeated randomised cross-over design. The randomisation was determined using a random number generator. Each rice sample was consumed on separate days one week apart from the previous study day to minimise any possible ordering effect [24]. Each day, participants reported at 6 a.m., after a 10 h overnight fast, to the Glycaemic Index Laboratory, Science Building One, University of Otago, Dunedin, New Zealand. Participants were advised to continue their usual daily activities without engaging in vigorous exercise or consuming food containing high fat, high sugar, or alcohol during the study period.

In each study visit, participants completed a series of three separate tests on one rice product: blood glucose responses test, satiety and palatability VAS, and chewing test. Fasting blood glucose was measured twice. Participants then consumed the rice sample (140 g) and a glass of water within 15 min following the fasting blood glucose measurement. Participants were asked to consume the rice samples at their normal chewing speed. Postprandial blood glucose concentrations were measured at 0, 15, 30, 45, 60, 90 and 120 min (HemoCue analysers; HemoCue^®^ Hb 201 System, Aktiebolaget Leo, Helsingborg, Sweden). Analysers were calibrated using an instrument-matched calibration cuvette, and checked for accuracy and reliability using three concentrations of control solutions (2.0 mmol/L, 4.5 mmol/L and 8.0 mmol/L) provided by the HemoCue manufacturer.

Satiety was recorded by participants at 0 (before rice consumption), 30, 60, 90 and 120 min using a modified version of a validated VAS. Participants were provided with eight satiety questions (“How hungry do you feel?”, “How satisfied do you feel?”. “How full do you feel?”, “How much do you think you can eat?”, “Would you like to eat something sweet?”, “Would you like to eat something salty?”, “Would you like to eat something savoury?”, “Would you like to eat something fatty?”). Participants were asked to rate five palatability questions on the visual appeal, smell, taste, aftertaste and overall palatability within 5 min immediately after rice consumption. For each question, participants were asked to mark their relative response on the unmarked visual analogue score line (10 cm in length). After completion of each questionnaire, it was turned face down on the desk to prevent participants from reviewing the previous results. The VAS score was analysed by measuring the length in cm (±0.1 cm) from the left end of the scale to the mark.

At the end of the testing (120 min), participants were given a tablespoonful of rice sample (10.6 ± 0.1 g) and asked to chew it at their normal chewing speed as they did previously in the glucose response test. Instead of swallowing, they were asked to expectorate the rice into a labelled container. Time (s) spent on chewing was recorded by the researcher. A sip of lukewarm water was then given to the participants to rinse the mouth and then expectorate the remaining chewed particles in the mouth into the same container. They were asked to repeat the chewing test if they accidently swallowed part of the rice sample. Expectorated samples were washed through a set of three stainless steel laboratory sieves with mesh apertures of 2000 μm, 1400 μm and 425 μm. The rice particles retained on the sieves and in the final wash were carefully washed (to eliminate the salivary α-amylase residues), collected and placed into metal dishes for drying in a convection oven at 70 °C and weighed twice at 24 and 48 h to ensure complete drying. Non-expectorated duplicate samples of cooked rice were used to determine moisture content. The particle size of the rice masticated by the 28 individuals was weighed into four ranges (over 2000 μm, between 2000 μm and 1400 μm, between 1400 μm and 425 μm, and less than 425 μm). For the weight of the dried sample and the moisture content, the proportions of the rice sample that passed through the sieves (“particle diameter > 2000 μm”, “particle diameter ≤ 2000 μm and >1400 μm”, “particle diameter ≤ 1400 μm and >425 μm”, and “particle diameter ≤ 425 μm”) were calculated. For each diameter, the same sieve was used each time to minimise cumulative errors and reduce variability. These particle sizes were chosen based on a previous study carried out in the same laboratory as this investigation [16].

### 2.5. Statistical Analysis

The incremental area under the blood glucose curve (iAUC) and the total area under the curve for each satiety parameter were calculated using the trapezoidal rule [4]. The finger prick blood glucose concentrations and area under the glucose response curve (AUC) were compared among three rice samples by repeated measurement analysis of variance (ANOVA). The chewed particle size distributions, chewing time, satiety, and palatability scores were also analysed by repeated measurement ANOVA. Univariate regression models with a random effect for participants were used to examine the association between the AUC for rice and variables including age, sex, BMI, chewed particle distributions, chewing time, satiety, and palatability scores. The regression analysis was also undertaken with the particle size distribution (%, the proportion of chewed rice particles) as the dependent variable and the chewing time (s) as an explanatory variable. The comparison for each expectorated rice sample was investigated across four ranges of rice particle size: ‘>2000 μm’, ‘<2000 μm and >1400 μm’, ‘<1400 μm and >425 μm’, and ‘<425 μm’. All statistical analyses were performed using SPSS version 12.0 (SPSS Inc., Chicago, IL, USA).

## 3. Results

### 3.1. Participants

All 28 volunteers completed the three study visits. The participants included 18 females and 10 males, aged 22.1 (95% CI: 20.0, 24.2) and 25.3 (95% CI: 20.7, 29.9) years respectively. Twenty participants were European, six were South Asian, and two were Chinese. In the screening session, all participants had normal fasting blood glucose (male, 5.0 (95% CI: 4.8, 5.2) mmol/L; female, 4.9 (95% CI: 4.8, 5.0) mmol/L) according to the World Health Organisation classification (normal fasting blood glucose less than 6.1 mmol/L).

By design, half the participants were normal weight (BMI < 25) or overweight including obese (BMI ≥ 25 kg/m^2^). No significant difference was found between the two BMI groups in fasting blood glucose (*p* = 0.589).

### 3.2. Comparison of Glycaemic Response Trajectory and the Incremental Area Under the Blood Glucose Response Curve (iAUC)

Among all experimental treatments, the fasting blood glucose concentrations were similar prior to consumption of each of the three rice meals (freshly cooked medium-grain white, 4.9 (95% Confidence Interval (CI): 4.8, 5.1) mmol/L; freshly cooked parboiled rice, 5.0 (95% CI: 4.8, 5.1) mmol/L; reheated parboiled rice, 4.8 (95% CI: 4.7, 5.0) mmol/L; Table 1) and were not significantly different (F = 1.020, *p* = 0.356). (Table 1)

**Figure 1:**
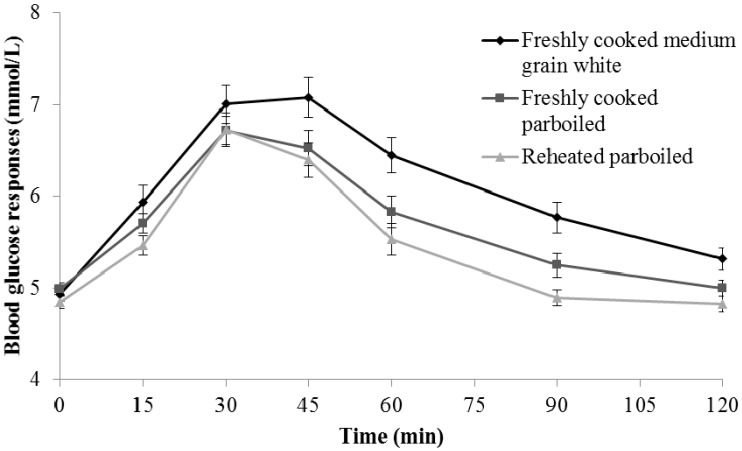
Postprandial blood glucose responses (means ± standard error) after consuming three rice samples (140 g): Freshly cooked medium-grain white (control), freshly cooked parboiled (Test 1), and reheated parboiled rice (Test 2). Test of homogeneity of variance had *p* = 0.001 at 15 min and 0.014 at 90 min).

Significant differences were observed in the iAUC for the three rice treatments (F = 9.555, *p* < 0.001). iAUC of the control rice, freshly cooked medium-grain white rice, was the highest: 1.7-fold higher (1.2, 2.6) than reheated parboiled rice (*p* < 0.001) and 1.5-fold higher (1.0, 2.2) than freshly cooked parboiled rice (*p* = 0.001). (Table 1, Figure 1). No statistically significant difference in iAUC was found between the reheated and freshly cooked parboiled rice (*p* = 0.445).

This initial faster and prolonged higher glycaemic response for the medium-grain white rice compared to reheated parboiled rice was sustained over 120 min. In contrast, the mean glycaemic response to reheated parboiled rice dropped below 5.0 mmol/L 90 min after consumption finished. (Figure 1) The repeated measures ANOVA test showed that the mean incremental blood glucose concentration (mmol/L) was significantly lower in reheated parboiled rice compared to freshly cooked medium-grain white rice at 15 min (−0.4 mmol/L, *p* = 0.015), at 45 min (−0.5 mmol/L, *p* = 0.018), at 60 min (−0.8 mmol/L, *p* = 0.001), at 90 min (−0.8 mmol/L, *p* < 0.001), and at 120 min (−0.4 mmol/L, *p* = 0.001). With increasing time, there was increasing divergence in the response curves by rice product: from around 15% (eta = 0.157 at baseline) to around 46% (eta = 0.459 at 90 min). The same test showed statistically significant differences between freshly cooked parboiled and freshly cooked medium-grain white rice at 45 min (−0.6 mmol/L, *p* = 0.050), at 60 min (−0.7 mmol/L, *p* = 0.020), at 90 min (−0.5 mmol/L, *p* = 0.007), and at 120 min (−0.4 mmol/L, *p* = 0.028) (Table 1). Overall, no significant difference was found between freshly cooked and reheated parboiled rice (both least significant difference (LSD) and Bonferroni tests *p* > 0.05).

### 3.3. Chewing Time and Proportions of Particle Size Distributions

The majority of the chewed rice was either smaller than 425 μm (~47%) or larger than 2000 μm (~44%) (Table 2). Chewing time and particle sizes varied widely among individuals but insignificantly among treatments and within individual (F = 2.966; *p* = 0.057). The average chewing time required for reheated parboiled rice (33.1 s (95% CI: 32.1, 34.1)) was 6.3 s longer than it was for the freshly cooked medium-grain white rice (26.8 s (95% CI: 26.3, 27.3), *p* = 0.026) and 5.3 s longer than freshly cooked parboiled rice (27.8 s (95% CI: 27.2, 28.4), *p* = 0.723). However, the only statistically significant difference (*p* = 0.026) was between reheated parboiled and freshly cooked medium-grain white rice. Chewing time (s) was negatively correlated with glycaemic responses (iAUC) for all rice samples (β coefficient = −2.360 (*p* = 0.089) for freshly cooked medium-grain white rice, β coefficient = −1.076 (*p* = 0.055) for freshly cooked parboiled rice, and β coefficient = −0.946 (*p* < 0.001) for reheated parboiled rice).

For each rice sample, the proportion (%) of the small size particles (between 2000 μm and 425 μm) was positively correlated with chewing time (s) (coefficient = 337.4 for freshly cooked medium-grain white rice (*p* < 0.001), coefficient = 248.2 for freshly cooked parboiled rice (*p* = 0.001), and coefficient = 298.2 for reheated parboiled rice (*p* = 0.008)) (Table 3). The proportions of big particle size (over 2000 μm) of reheated parboiled rice were significantly inversely correlated with chewing time (coefficient = −43.6 (*p* = 0.005)), while no significant correlations were found in freshly cooked parboiled and medium-grain white rice (Table 3).

### 3.4. Satiety and Palatability Responses (VAS Scores) over 120 min

**Figure 2:**
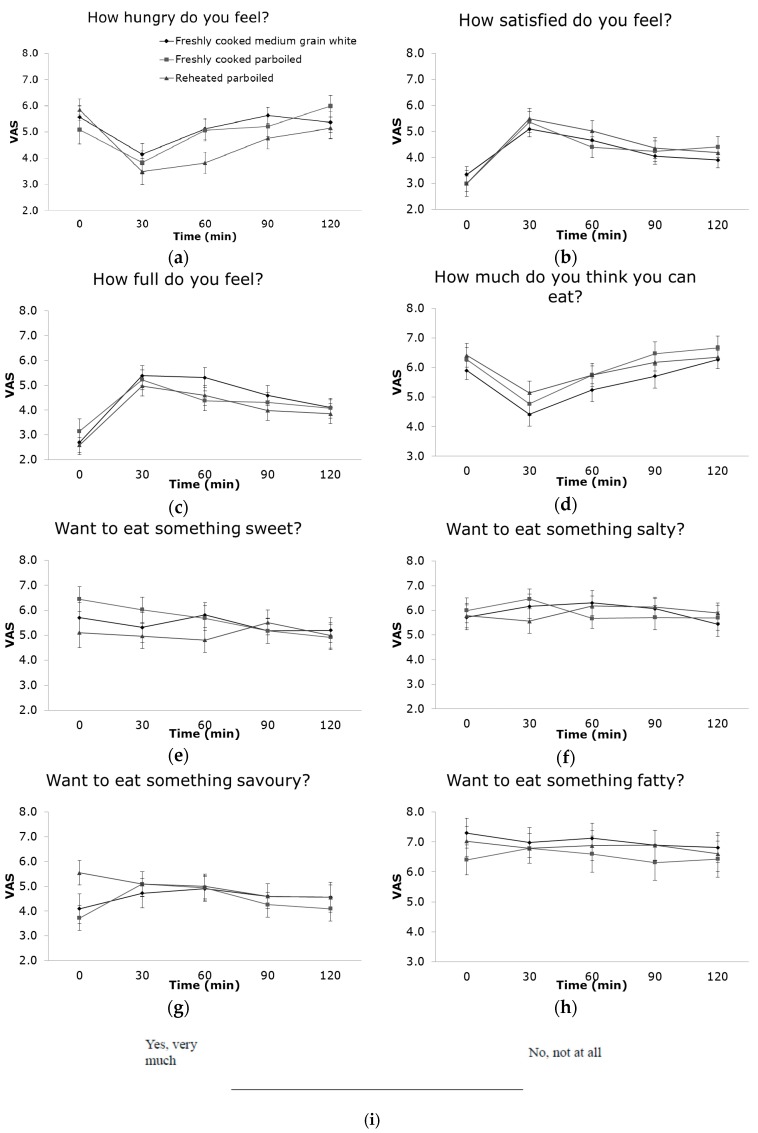
Mean satiety scores (VAS, visual analogue scale scores) change over 120 min for freshly cooked medium-grain white rice, freshly cooked parboiled rice and reheated parboiled rice, and each satiety VAS question: (**a**) “How hungry do you feel?”; (**b**) “How satisfied do you feel?”; (**c**) “How full do you feel?”; (**d**) “How much do you think you can eat?”; (**e**) “Want to eat something sweet?”; (**f**) “Want to eat something salty?”; (**g**) “Want to eat something savoury?”; (**h**) “Want to eat something fatty?”; (**i**) Line scaling for measuring participants’ satiety on a 10 cm visual analogue scale (VAS). Error bars show the standard error of the mean VAS score.

The satiety responses to the three rice samples were not significantly different. However, participants who had reheated parboiled rice felt hungry approximately 30 min later than the other two freshly cooked rice products (medium-grain white and parboiled; F = 3.281, *p* = 0.043) (Figure 2a). At 60 min, the VAS scores of “How hungry do you feel?” for reheated parboiled rice was 1.3 cm (95% CI: 0.16, 2.4) lower than freshly cooked medium-grain white rice (*p* = 0.026) and 1.2 cm lower than freshly cooked parboiled rice (*p* = 0.033). No other significant differences for the desirability of sweet, salty, savoury, fatty, or other satiety questions were found (Figure 2). Simple linear regression analysis showed no association between any of the mean satiety scores and the blood glucose concentration, nor between satiety and chewing.

**Figure 3:**
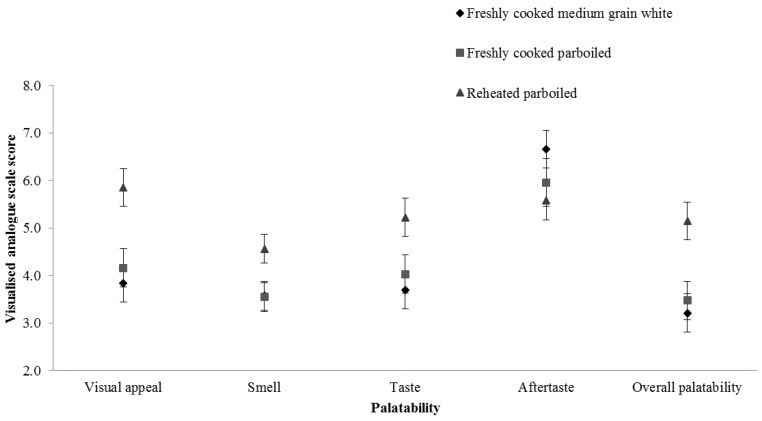
Mean palatability scores (VAS, visualised analogue scales scores) immediately after finishing eating prepared control and test rice samples (freshly cooked medium-grain white rice, freshly cooked parboiled rice and reheated parboiled rice) for each palatability VAS question.

Reheated parboiled rice showed generally higher palatability scores than the other two freshly cooked meals (Figure 3). The visual appearance of reheated parboiled rice score was around 2.0 times higher than freshly cooked medium-grain white rice (*p* < 0.001) and 1.7 times higher than freshly cooked parboiled rice (*p* = 0.003). The smell of reheated parboiled rice was preferred to the other two freshly cooked rice meals (around 1.0 times higher than the control rice (*p* = 0.034) and around 1.0 times higher than the freshly cooked parboiled rice (*p* = 0.029)). The parboiled rice taste scored around 1.5 times higher than freshly cooked medium-grain white rice (*p* = 0.023). However, no significant differences were found in the palatability of the aftertaste. Overall, reheated parboiled rice was preferred compared with medium-grain white rice (*p* = 0.003) and freshly cooked parboiled rice (*p* = 0.012).

## 4. Discussion

Twenty-four hours’ cold storage and then reheating of cooked parboiled rice resulted in 1.7-fold (*p* < 0.001) lower blood glucose concentration trajectories over two hours than freshly cooked medium-grain white rice. Chewing time for 10 g cold-stored parboiled rice was 6 s (25%) longer and was considered more palatable, visually appealing and better tasting than freshly cooked medium-grain rice (all *p* < 0.05).

Others have reported a reduction in glucose trajectory with extent of parboiling [14] and consumers reheating cooked rice [25]. The role of the parboiling and cold storage in relation to reduced glycaemic response may be explained by the considerably higher proportion (approximately 30% higher) of RS in reheated parboiled rice than in freshly cooked medium-grain white rice reported in an in vitro experiment [12]. Parboiling pre-treatment and cold storage preparation method are associated with specific changes to the physico-chemical properties of the rice (i.e., rice starch retrogradation that increased the amylopectin and amylose crystallisation) and thus reduced the starch digestibility [26] and glycaemic responses [27]. It is suggested that cold storage significantly increased the amylose and amylopectin crystallisation [10] and enhanced its resistance to digestion [11] although the difference in the glycaemic trajectory for parboiled rice between fresh and reheated was not statistically significant.

The effect of cold storage and reheating on reducing glycaemic responses of parboiled rice was not significant in this study compared with freshly cooked parboiled rice. The systematic review by Boers et al. [27] indicated that multiple heating–cooling cycles can achieve a high RS content. In this study, only one heating–cooling cycle was applied. Boers et al. [25] also indicated that increasing RS in rice varieties by up to ten times did not have an effect on GI. The review also suggested that the change in RS content following heating–cooling cycles was insufficient (possibly due to the small differences in amylose content in rice varieties) to observe a significant change in glucose response or GI [25]. Also, the reheating (to 65 °C) might have reversed the retrogradation by breaking some of the amylopectin crystallites [12], and thus, lessening the impact on any possible reduction in glycaemic responses.

A mouthful of reheated parboiled rice was chewed for 6 s longer than the freshly cooked rice and there was a significant negative correlation between the proportion of large particle size (> 2000 μm) and chewing time. Although the degree of habitual mastication and the time of chewing required for preparing a food suitable for swallowing varied considerably among participants, they were relatively constant within individuals as previously reported [16]. The degree of habitual mastication (i.e., particle sizes) and the chewing time required for mastication depends on the food type, the saliva flow and the ability to form a cohesive, liquid coated bolus that can be swallowed [28]. Masticated rice with a higher proportion of RDS has been reported to form a bolus more easily than those with a lower proportion of RDS [16]. Cold storage promoted the retrogradation of rice starch (i.e., higher proportion of RS) and increased the hardness of the rice grain (i.e., change in texture after parboiling and cold storage), which, in turn, would prolong the chewing time for each mouthful [12].

As hypothesised based on the in vitro results [12], thorough mastication (i.e., increased chewing time) breaks rice grains into smaller particle sizes, increases the mixing of rice particles with salivary enzymes, improves hydrolysis of starch in mouth and stomach and, thus, is expected to increase the acute glycaemic response. Surprisingly, an inverse association between chewing time and postprandial glucose response was found. Others have also found that a longer chewing time is associated with the slower glycaemic response [18,29]. In 2005, Suzuki et al. [30] found that the insulin-impaired participants showed a greater glycaemic response after shorter chewing time compared with participants with normal insulin tolerance and extended chewing time, which confirmed that the degree of chewing might have a direct and positive effect on the rate of in vivo digestion and the glycaemic response.

One theory is that increasing chewing time can increase both the time of the food in oral cavity and oral amylase action [18,29]. It was proposed in some studies that the glucose release from starch digestion in oral cavity might also induce vagal activation and result in early insulin secretion (i.e., preabsorptive insulin or cephalic phase of insulin secretion) and thus reduced glycaemia [29]. Another theory is that more thorough mastication (i.e., longer chewing time) and swallowing a food bolus with fine particles (<2000 μm) may delay gastric emptying and thus reduce postprandial glycaemia [6,29,31]. A bolus with finer particles reduces the viscosity of gastric content and increases pyloric outflow, thus increases sedimentation of solids in liquids, and promotes the ability of the antrum to preferentially empty liquids faster than solids [28]. The increasing interaction between macronutrients, such as starch molecules, amylopectin, and amyloses, and the receptors within the small intestine, promotes the stimulation of the vagus nerve and the secretion of insulin and gut hormones (GLP-1, CCK, and PYY) from entero-endocrine cells while suppressing glucagon, shortens the subsequent gastric emptying, and thus, reduces postprandial blood glucose [31].

Few studies have investigated the effect of rice with differenct starch digestibility profiles on appetite. Zenel et al. found that participants were significantly more hungry at 30 min after high amylose rice with higher RS content compared to low amylose rice with lower RS [32]. In a study by Chiu et al., it was reported that there was no significant difference in appetite between short-grain rice and high RS rice in healthy adults [33]. In this study, the only significant difference in appetite scores was observed at 60 min, when the participants reported feeling less hungry after consuming reheated parboiled rice than after freshly cooked parboiled and medium-grain white rice. A recent systematic review further suggested that longer chewing may decrease self-reported hunger and possibly alter the gut hormone response related to satiety [34]. However, we did not find an effect on satiety of reheating the parboiled rice compared to the other two rice samples despite the significantly different iAUC of blood glucose responses. Participants preferred the reheated parboiled rice compared to the other two samples for visual appeal, smell and taste.

One strength of this study is that it followed the cold storage and reheating method from the New Zealand Food Safety Authority guidelines, which has indicated the safe practice of preparing boiled rice to stop *Bacillus cereus* growth during storage and stop toxin production. The cooked rice may be kept for a short time at more than 60 °C or cooled quickly and stored for up to one day [35]. The Ministry for Primary Industries [19] advised that cooked rice should be removed from the hot container and spread into a clean shallow container (<10 cm deep) and then placed in a refrigerator at 4 °C or lower to be cooled quickly and evenly to prevent bacteria growth and toxin production. It is required to evenly reheat the cold-stored rice to over 65 °C to destroy the bacteria and production of toxins.

Another strength of this study is that it followed a glycaemic response method whose precision and validity were tested and validated by previous studies. The group of participants consumed a serving of each rice sample on separate experiment days (seven days apart) to minimise possible intra-individual variation and to improve precision. On each experiment day, the volumes of the test meals were similar, the time spent on consuming the meals was restricted to less than five minutes, all rice meals were fully gelatinised, and the rice grains all retained their original shape when they were served to participants to minimise the influences on glycaemic response and satiating power of each rice meal.

Limitations of the study include that each condition, including the chewed sample, was measured only once. Participant compliance was high and all participants completed all measurements. However, this relatively small study is only applicable to these specific brands of rice cooked in this specific way. It should be acknowledged that the volunteers may not necessarily be representative of the population at large that some unrecognized attributes of this convenience sample could explain the marked differences in glycaemic responses among three rice samples. Variation among the brands or rice (such as rice origin, storage time/condition, and humidity) and the methods of cooking also needs to be explored further with more participants to be powered to find further influences. The two factors that might have affected the gastric emptying rate, glycaemic response, and the feelings of satiety and palatability are the chewed particle size and the net energy of the rice meals served to each participant. Although chewed particle size is impossible to control, the net energy may be monitored by dietary measurements in order to explain changes in the subjective satiety score in the future.

## 5. Conclusions

Compared with freshly cooked white rice, reheated cold-stored parboiled rice and freshly cooked parboiled rice reduced the postprandial glycaemic response and extended the chewing time. No significant difference in postprandial glycaemic response was observed between freshly cooked and reheated parboiled rice samples. The reheated parboiled rice was preferred due to improved palatability. For regular consumers of parboiled rice, preferably reheating cooked rice after cold storage would lower the dietary glycaemic load and, in the long term, may reduce the risk for type 2 and gestational diabetes. More trials are needed.

## Figures and Tables

**Table 1:** Means of blood glucose responses (mmol/L) at baseline and incremental blood glucose response (mmol/L) at each time point after consuming three rice samples (140 g) and the incremental area under the glucose responses curve (iAUC) (mmol/L·min).

Rice Samples		Incremental Area Under the Curve (iAUC)	Baseline Blood Glucose	Incremental Blood Glucose Responses (mmol/L)					
**Cooking Methods**	**Rice Products**	**(mmol/L·min)**	**(mmol/L)**	**15 min**	**30 min**	**45 min**	**60 min**	90 min	120 min
**Control Rice**									
Freshly cooked	Medium-grain white	144.7 ^a^ (119.8, 169.6)	4.9 (4.8, 5.1)	1.0 ^a^ (0.7, 1.3)	2.1 (1.7, 2.5)	2.1 ^a^ (1.7, 2.5)	1.5 ^a^ (1.1, 1.9)	0.8 ^a^ (0.5, 1.1)	0.4 ^a^ (0.1, 0.6)
**Test Rice**									
Freshly cooked	Parboiled	94.9 ^b^ (75.5, 114.4)	5.0 (4.8, 5.1)	0.7 (0.5, 0.9)	1.7 (1.4, 2.0)	1.5 ^b^ (1.2, 1.9)	0.8 ^b^ (0.5, 1.2)	0.3 ^b^ (0.0, 0.5)	0.0 ^b^ (−0.1, 0.2)
Reheated	Parboiled	83.5 ^b^ (63.4, 103.6)	4.8 (4.7, 5.0)	0.6 ^b^ (0.4, 0.8)	1.9 (1.5, 2.3)	1.6 ^b^ (1.2, 2.0)	0.7 ^b^ (0.3, 1.1)	0.0 ^b^ (−0.1, 0.2)	0.0 ^b^ (−0.2, 0.2)

Note: 95% confidence intervals (CI) for each mean of blood glucose responses (mmol/L) are in brackets. The means in the same column with the different letter are significantly different (*p* < 0.05).

**Table 2:** Before-swallowing rice particle size distribution (%) and chewing time (s). (*N* = 28) Values are mean and standard deviation, unless otherwise stated.

Masticated Particle Size	Freshly Cooked Medium-Grain White	Freshly Cooked Parboiled	Reheated Parboiled
**Particle Size Distributions (% by Weight)**	**Mean (95% CI)** **[Range of Particle Sizes (%)]**		
>2000 μm	46.1 (44.9, 47.3) [0.5–71.2]	44.0 (43.1, 44.9) [21.4–74.1]	42.6 (41.4, 43.8) [17.2–83.6]
<2000 μm~>1400 μm	2.9 (2.8,3.0) ^a^ [0.3–5.8]	4.4 (4.3, 4.5) ^b^ [1.5–7.7]	4.9 (4.7, 5.1) ^b^ [0.9–8.5]
<1400 μm~>425 μm	3.1 (3.0, 3.2) ^a^ [1.2–6.1]	4.6 (4.4, 4.8) ^b^ [1.4–9.4]	5.3 (5.1, 5.5) ^b^ [0.3–12.6]
<425 μm	47.9 (46.8, 49.0) [25.0–96.7]	47.0 (46.1, 47.9) [17.0–73.0]	47.8 (46.6, 49.0) [10.9–70.8]
**Chewing time (s)**	**Mean (95% CI)**		
	26.8 (26.3, 27.3) ^a^	27.8 (27.2, 28.4)	33.1 (32.1, 34.1) ^b^

ANOVA test. *p*-value with * (<0.05) indicates that the mean in the same row is significantly different from others. The means with the different letter in the same row indicates a significant difference between these two means (*p* < 0.05).

**Table 3:** Correlations between particle size distributions (%) (as dependent variable) and chewing time (s) (as explanatory variable) (*N* = 28) by multiple linear regression analysis.

Rice Sample Particle Size (μm)	Coefficient	Constant	R	F-Value	*p*-Value ^1^
**Medium-grain wite**	**Freshly cooked medium-grain white**				
>2000 μm	−13.985	33.197	0.307	2.701	0.112
<2000 μm~>1400 μm	168.442	21.859	0.367	4.057	0.050 *
<1400 μm~>425 μm	337.431	16.178	0.649	18.871	<0.001 *
<425 μm	10.365	21.789	0.220	1.326	0.260
**Test rice 1**	**Freshly cooked parboiled**				
>2000 μm	−32.266	41.963	0.435	6.052	0.021
<2000 μm~>1400 μm	309.665	14.204	0.624	16.555	<0.001 *
<1400 μm~>425 μm	248.154	16.396	0.583	13.373	0.001 *
<425 μm	16.946	19.785	0.234	1.504	0.231
**Test rice 2**	**Reheated parboiled**				
>2000 μm	−43.571	51.654	0.516	9.449	0.005 *
<2000 μm~>1400 μm	112.205	28.286	0.149	0.592	0.449
<1400 μm~>425 μm	298.233	17.178	0.490	8.197	0.008 *
<425 μm	40.906	13.557	0.457	6.880	0.014 *

^1^ ANOVA test for multiple linear regression and residue. *p*-value with * (<0.05) indicates there is a significant linear relationship between particle size distribution and chewing time (s).
